# A qualitative study of National Health Service (NHS) complaint-responses

**DOI:** 10.1186/s12913-021-06733-5

**Published:** 2021-07-15

**Authors:** May McCreaddie, Bethan Benwell, Alice Gritti

**Affiliations:** 1 School of Nursing and Midwifery, Royal College of Surgeons in Ireland, Medical University of Bahrain, PO Box 15503, Adliya, Kingdom of Bahrain; 2 Faculty of Arts and Humanities, University of Stirling, Dalkeith, Scotland, UK; 3 Newbattle Abbey College, Dalkeith, Scotland, UK

**Keywords:** Healthcare complaints, Complaint resolution, Qualitative study, Complaint-responses, Rhetoric, Discourse analysis, Complaint handling

## Abstract

**Background:**

Healthcare complaints are grievances that may be indicative of some system failures, individual failings, or a combination of both. Moreover, the experience of making a complaint, including its outcome, often falls short of patient expectations, particularly in relation to the interpersonal conduct of National Health Service (NHS) staff. Over half of unresolved (local) complaints are subsequently upheld by the ombudsman with others potentially resulting in costly litigation.

**Method:**

A nuanced discourse analytical approach to analysing the language choices within complaint-responses could potentially provide greater insight into why many local complaints continue to remain unresolved. Over a period of 1 month we collated a data corpus of written complaints and their responses (*n* = 60) from an NHS healthcare area in Scotland, United Kingdom (UK) following anonymisation by NHS complaint handling staff. We took a qualitative approach to analysing the data drawing upon Discourse Analysis with this paper reporting on the complaint-responses only (*n* = 59). We had undertaken a similar review of the initial written complaints and this is reported elsewhere. In this paper we examine *how, and to what extent*, the complaint-responses fully addressed the complainants’ perceived grievances.

**Results:**

The complaint-responses rarely acknowledged the amount of detail or ‘work’ involved in making the complaint. Complaint-responses constructed complainants’ accounts as subjective by using specific discourse strategies. Further, complaint responses used unintentionality or exceptionality to mitigate sub-standard experiences of care. We also observed the ‘fauxpology’ - a non-apology or false apology (e.g. I am sorry *you feel*) which imputes the cause of distress to the subjective (and possibly misguided) impressions of the complainant. The complaint-responses thereby evade blame or responsibility for the complainable action by implying that the complainants’ feelings do not align with the facts.

**Conclusions:**

Complainants and complaint-responders work to different frames of reference. Complaint responders need to engage and align with complainants from the outset to ensure more appropriate complaint- responses. Complaint resolution as opposed to complaint handling could be enhanced by the approach of linguistic analysis and reference to the consumer literature’s justice-based approach to post-complaint behaviour.

## Introduction

The United Kingdom (UK) NHS is a unique institution established in 1948 which provides healthcare for all based upon need, not ability to pay. Funded by taxes, its consumers therefore, view themselves as its custodial guardians with attendant high expectations of the healthcare it provides. Yet complaints about the National Health Service (NHS) are consistently prevalent if not increasing [1, 2]. Complaints are grievances that may be indicative of some system failures or individual failings, or a combination of both [3, 4] and are therefore, representative of problems concerning individuals and, or their organisation. Moreover, the experience of making a complaint, including its outcome, often falls short of patient expectations [5–7], particularly in relation to the interpersonal conduct of NHS staff [8, 9]. This is highly consequential for the NHS because litigation can result from dissatisfaction with, and exhaustion of a system not meeting a service user’s needs [10]. Litigation is costly to complainants’ health and wellbeing and the NHS budget [11]. Moreover, the strongest predictor for litigation is not medical error but dissatisfaction with communication, either within the clinical encounter [12] or subsequently in the complaints handling process [13, 14].

## Background

Complaints may be partly indicative of the public’s supposedly unrealistic expectations of ‘their’ NHS [15, 16] expressed as a general lack of patient-centeredness if not ‘clinical heartlessness’ ([17]: 956). Complaints may also provide ‘insights’ into patient experience for example, but they are not considered patient safety red flags per se [3, 18, 19]. Nevertheless*,* they clearly indicate a broader conceptualisation of ‘harm’ [20] and could play a role in improving quality [18].

NHS complaints are heterogenous [21], relatively unstructured [3], complex and emotive [22, 23] with many evidencing serial failings [3] that subsequently breach a given threshold of patient satisfaction with care [24] and the point at which patients and, or their relatives, are more likely to formally complain. Complainants often report psychological distress throughout the whole process from care to complaint - as well as with the subsequent outcome, which may affect their view of future healthcare [5, 25]. The Parliamentary and Health Services Ombudsman in England recently undertook research into the management of healthcare complaints and concluded that complaints, rather than providing opportunities for learning or patient involvement, are still negatively perceived by healthcare entities. Further, there were considerable variations in what constitutes good practice in complaint-responses with disparate training provided for complaint handlers [26].

NHS complaints are a significant problem that, at best, highlight disaffection with a treasured institution. What is perhaps most concerning about the number of NHS complaints is that over half of unresolved (local) complaints are subsequently upheld by the ombudsman. Moreover, the Francis Report [9] - the public enquiry into poor care, unnecessary patient deaths and the failure to heed warning signs of serious failings at the Mid Staffordshire NHS Foundation Trust in England – also suggested that NHS complaint handling as opposed to complaint resolution is perhaps not as robust as it should be.

We believe a nuanced linguistic approach to analysing complaint-responses could potentially provide greater insight into why many local complaints continue to remain unresolved. We therefore, reviewed a data corpus (*n* = 60) of written complaints and their responses collated over the period of 1 month from an NHS board area in Scotland, UK. In an earlier published study, we aimed to provide a typology of complaints, complainants and *how* they complain and this is reported elsewhere [3]. In this paper we review the complaint-responses only (*n* = 49). We examine complaint handling via the complaint-responses and therefore, *how, and to what extent*, the complaint-responses address the complainants’ perceived grievances.

## Methods

We took a broad qualitative approach drawing upon Discourse Analysis (DA). DA is the systematic study of text and talk and here we adopt the ethnomethodological version of Discourse Analysis that emerged from the work of Gilbert and Mulkay [27] (1984) and the rhetorical analysis of Billig [28] (1987). This approach was developed by social psychologists at Loughborough (e.g. Potter and Wetherell 1987) [29] and served as the foundations for the discipline of Discursive Psychology (Potter and Edwards 1992) [30]. Discourse Analysis within this tradition derives observations from data and the orientations of the participants (rather than imposing the concerns of the analyst on the data) and the analysis therefore, primarily focuses on the linguistic features of the discourse. It is a method that treats texts as social practices in which the construction and narrating of events is a way to manage and curate issues of blame, agency, responsibility, emotion, belief and attitude. We analysed how complainants and, in this paper, complaint-handlers attempted to present and accomplish certain actions with specific segments of data subject to an enhanced linguistic analysis attending to the concerns of DA as set out above e.g. extreme case formulations (ECFs), idioms, pronouns, shifts in footing, modality, hedging and reported speech. Our focus was therefore, not on understanding or behaviour but on what actions are being accomplished through the complaint-responses of the complaint-handlers [31].

The data corpus and the analytical approach allowed for a thorough review of the written discourse of complaint responses to enhance our understanding of how potential conflict is managed; how complaint handlers communicate their objectives clearly (or not) and last but not least, how emotion is ‘managed’ in these sensitive encounters.

### Ethics

All procedures and methods performed in this study involving human participants were in accordance all the relevant guidelines and regulations. Ethical approval was obtained from the Local Research Ethics Committee (Tayside Local Research Ethics Committee 11/ES/0048). All written complaints and their responses over the period of 1 month were collated and fully anonymized by the complaint handlers prior to being received by the researchers, negating the need for explicit a priori consent, as approved by the LREC. Caldicott data protection consent forms [32] were completed by the Principal Investigator with data securely held in storage (soft and hard copies) as per ESRC guidelines [33]. No subjects were aged 18 or under. No experimental protocols were involved in this study.

### Data analysis

We uploaded all data into a database (Bristol Online Survey: BOS) which outlined each complaint and complaint-response in detail (who, what, how, outcome etc). We also independently reviewed specific linguistic strategies e.g. ECFs, minimisers, mitigation, humour, repetition etc. - for each complaint and complaint-response. Thereafter, we placed the complaints and complaint-responses into two broad categories depicting the complexity of the complaint and its primary focus: complex care and treatment-related complaints (*n* = 42) and single-issue administrative complaints (*n* = 18).

Our data corpus comprised 60 letters in total obtained over the period of 1 month (Table 1). There were 49 paired complaints (complaint and complaint-response). In addition there were 10 complaint-responses (with no initial complaint) (*n* = 59). There was one additional complaint letter returned to the sender with an apology but refusing to action the complaint (*n* = 60). Thus, there were 59 formal complaint-responses in total. This article reviews the complaint-responses only.

**Table 1:** The data corpus of written complaints

Complaint Category	Number	Sub-totals
Paired Complaint: Complaint and Complaint Response	49	
Complaint only (letter returned)	1	50 complaints in total
Complaint response only (no complaint available)	10	59 Complaint-responses in total
**TOTAL**	**60**	

A primary reason for complaining is to acquire an outcome or judgement, preferably one that acknowledges the complaint, provides an explanation and offers some kind of redress [34]. Consequently, any decision or outcome is inextricably linked to the language choices, actions and therefore, how the complaint-responders account for and explain the decision. We therefore, reviewed in detail the complaint decision or outcome (complaint-response) as detailed below.

### Complaint outcome (*n* = 51/59)

A decision was specified (DS) in 28 of the total 59 complaint-responses: 16 upheld, 3 partly upheld, 9 not upheld (*n* = 28). In the remaining complaint-responses a decision was not specified (NS) (*n* = 31).

The categories of upheld, partly upheld and not upheld are common parlance in complaints handling and are used by the relevant agency to specify whether the standard of service was one that a reasonable person could expect. If the complaint is considered valid it is upheld, if not it is therefore, not upheld. The category of ‘partly upheld’ is evidently more problematic as it is not always clear which part is actually valid. Thus, the categories of upheld, partly upheld and not upheld are those ascribed by the complaint handlers (*n* = 28) within the complaint-responses.

The decision not specified (NS) category (*n* = 31) was therefore, further examined to see if a decision or ‘judgement’ could be ascertained on the basis of the reply content of the ‘decision-specified’ complaint-responses (*n* = 28). Accordingly, we then placed 22 (*n* = 31) of these NS complaints into one of the three institutionally-defined ‘judgement’ categories; upheld, partly upheld, not upheld.

Table 2 outlines the nature of complaint by outcome before and after the review discussed above.

**Table 2:** Nature of complaint and complaint-response/outcome

Nature of complaint	Upheld			Partly Upheld			Not Upheld			Complaint Response with Decision (DS or NS)	Unable to ascertain possible complaint-response from letter content and, or DS letters	All Complaints
	DS	NS	total	DS	NS	total	DS	NS	total			
**Single**	7	5	12	1	X	1	1	2	3	16	2	18
**Complex**	9	2	11	2	X	2	8	14	22	35	6	41
**TOTAL**	16	7	23	3	X	3	9	16	25	51	8	59^a^

DS = decision-specified in the complaint-response

NS = decision not specified in the complaint-response, but a decision outcome probable from reply content

^a^ excluding 1 complaint letter returned to the sender with an apology but refusing to action the complaint (*n* = 60 in total)

Once these broad patterns of complaint-responses were identified we were then in a position to relate language choices and actions to the decisions. A systematic DA approach to identifying language choices relevant to the act of justifying the decisions, explaining the actions of the relevant healthcare providers or offering apologies for the complained-about events or actions, was then applied using the BOS data corpus. In the results section below we provide a selective qualitative analysis of examples from our data in order to exemplify the role of discursive choices in formulating responses to complaints.

## Results

Our systematic DA approach highlighted a number of consistent patterns emerging across the complaint-responses and for the purpose of our analysis we focus on four of the most marked examples:

- Subjective patient accounts contrasted with objective staff accounts and treated as ‘not proven’ or ‘not upheld’ complaints.
- Actions by staff represented as ‘unintentional’ or ‘exceptional’ thus mitigating sub-standard care
- Incongruences: rebutting complaints but amending practice
- Negligence: opposing frames of reference

Seven out of 16 single-issue responses were upheld, rising to 12 out of 16 when the NS revised figures were added. However, only 9 out of 35 complex complaints were upheld, rising to 11 out of 35 when NS revised figures were added. In short, complainants were more likely to have a decision stated and upheld if they complained about a single-issue only and about administrative factors rather than care and treatment.

It is evidently easier to investigate one issue than it is to review a series or sequence of issues. Further, administrative issues tended to provide objective evidence (e.g. appointment times, staffing, car parking) as opposed to care and treatment issues which invariably involved different perspectives and in turn, arguably subjective accounts. Accordingly, complaint handlers may have been willing to admit to failings in impersonal, administrative aspects rather than the personal and fundamental parts of a national health service that is predicated on ‘care’. Finally, administrative failings are arguably less likely than care and treatment errors to attract costly legal proceedings.

### Complaint-responses

Complaints are involved narratives, providing considerable detail in chronological and cumulative accounts [3]. In contrast, the complaint-responses outlined here rarely acknowledged the amount of detail or ‘work’ involved in making the complaint as well as the significant personal context provided. Consequently, there was a lack of acknowledgement of (a) the emotion expressed in the complaint, (b) the harm reportedly experienced by the patient and, or those involved in the complaint plus (c) the time taken to formulate the complaint and (d) the cumulative distress potentially experienced by the complainant in so doing. The complaint handlers, therefore, demonstrated a lack of alignment to the complaint and complainant. In this instance, ‘alignment’ is used to refer to responses that cooperate at a structural level, that facilitate and support the activity or sequence and, or accept the presuppositions and terms of the proposed action or activity [35]. The lack of alignment from the outset perhaps set the tone for the responses that followed. We will now detail four key areas that were consistent across the complaint-responses.

#### Subjective patient accounts versus objective staff accounts = not proven or not upheld

All complaint-responses treated complainants’ accounts as being subjective and therefore, by implication, not necessarily factual. Complainants’ descriptions of events were presented in terms of perceptions and feelings – a particular view of events embedded in emotion. Conversely, staff accounts explicitly denied any wrongdoing and presented the contested events in relatively neutral terms. In addition, the complaint-responses used medical or nursing notes to corroborate the staff member’s account. Notably, the complainants may have already provided corroboration from various sources (e.g. relatives, other staff) yet this was not acknowledged in the response. Consequently, in the apparent absence of ‘evidence’, the complaint was dismissed in judicial terms (e.g. unable to substantiate, or ‘partly upheld’) with the part that was upheld not necessarily specified. In some instances - and aligning neatly with the Scottish judicial system - no outcome was stated and a ‘not proven verdict’ was therefore, inferred. [Scotland has 3 judicial verdicts: Guilty, Not Guilty and Not Proven – with latter largely unique and meaning the charge has not been corroborated and the accused is therefore, acquitted.] [36].

*“While I acknowledge and note* ***your*** *strong views I must advise that the staff do not share* ***your*** *viewpoint. Despite our enquiries into the matter I have been unable to substantiate the concerns* ***you*** *have alleged. [3984)”*

‘*We sincerely apologise if* ***your experience*** *as a patient within X was not a positive one’. It is always disappointing to us when patients feel the service* ***they*** *have received falls below the standards* ***they*** *expect’.[3971].*

NB: the text in bold and underlined is our emphasis and directly relates to the analysis that follows or preceeds the text.

There are a number of aspects to the specific strategy of footing (i.e. the alignment of communication between the complainant and the complaint-responder) and this evident in all of the above excerpts. The use of pronouns throughout (specifically ‘you’) repeatedly denotes the complaint-responder as occupying a different perceptual stance to that of the complainant. This contrast in stance also enables the complaint-responder to index how they are likely to manage the complainant’s experiences and expectations (i.e. formally and potentially non-aligned) [36]. Finally, by specifying ‘your experience, they expect’ the complaint-responder is selectively accrediting the emotive account of the patient’s experience to the individual subject. Accordingly, the complainant’s reported emotions can then be treated as less factual and perhaps, therefore less important.

Minimisers (‘not a positive one’) were also used to mitigate an experience and therefore, reduce its significance. Often this is achieved by expressing a depreciatory proposition or utterance that denies the complainant claim, and then proceeds to affirm a different process [37]. This is particularly evident in the following excerpt [3921] where a husband complained on behalf of his wife who had reportedly felt ‘*humiliated and demeaned’* during a consultation.

*‘****You felt*** *the consultation did not go as planned’ (footing, minimisers)*

*‘The consultation was undertaken with care and attention’ (refers to‘objective’ medical records).*

The complainant’s expression of ‘*humiliated and demeaned’* is not repeated within the complaint-response but instead, is minimised to ‘*did not go as planned’* which is subsequently contrasted with an alternative account of a consultation undertaken ‘*with care and attention’*, with the latter appearing to have been corroborated from medical notes.

By ascribing a subjective stance to the complainants’ grievances and through comparing and contrasting this with the minimal or more neutral statement provided by staff and, or patient records, the complaint-responder provides the background for their rebuttal/part rebuttal of the complaint. Notwithstanding the verdict, the complaint-responder now provides a rationale as to why any unwarranted acts may have occurred.

#### Unintentional or exceptional: mitigating sub-standard care

Notably in many of the replies the complaint-responder stated that any possible transgressions were unintentional on the part of the staff member.

‘*Staff* ***would not knowingly*** *prevent any patient from having pain relief and are sorry if at any time this was X experience’ (3984)*

*‘This was not* ***his (Doctor’s) intention’****, [3921]*

Furthermore, having apparently established unintentionality on the part of the staff member, the complaint-responder arguably goes beyond this through the positive approbation of staff’s motivation whose behaviour was the focus of complaint.

*Although X acted with the* ***best of intentions*** *I am sorry for the upset you experienced [3934]*

In short, the complaint-responder ascribes a moral incentive to the actions of the staff involved, thereby effectively closing down further criticism. Having established unintentionality, a number of complaint-responses also cited exceptionality as a possible mitigating factor [38]. Exceptional circumstances include the illegitimate stressors of healthcare [39] - those outside the control of the staff member (e.g. workload, throughput, inexperience, other patients, multi-tasking):

*(Hospital was) ‘particularly busy’ [3956]*

*(Hospital was) busier than normal’ ‘locum relatively inexperienced’ ‘queueing’ ‘other patients’ [3965]*

*‘Staff need to prioritise care constantly’ ‘unpredictability of their workload’ [3984]*

Furthermore, it is sometimes implied that patients themselves somehow contributed to the contested event:

*(You are a) high risk for major surgery’ (3962)*

*Your case had been difficult from the start’ ‘specialist equipment … [was] hard to come by’ [3958]*

*‘However an ankle fracture booklet was not given because you had a ligament reconstruction which is very uncommon’ (3977)*

Illegitimate stressors and unintentionality may combine and, in the view of the complaint-responder, simply be misinterpreted by the complainant:

*Out of Hours (OOH) was busy, (the doctor) wanted to examine quickly for urgent treatment – his sense of urgency came across as rudeness’ [3963]*

Finally, if unintentionality or exceptionality could not be established then a less common strategy appeared to be simply to state that there had been ‘*no previous complaints’* per se [3927]. Thus, the veracity of the complainant’s account is further contested.

#### Incongruences: rebutting complaints but amending practice

Non-administrative complaints tend to occur once a (satisfaction) threshold is breached and at this juncture the complaint is a usually series of unresolved repeated grievances, making the complaint-response more challenging. However, there appeared to be a number of incongruities in the complaint-responses. Seven complaints were largely rebutted, but then included some detail on numerous changes that had been made as a result of the complaint. Whilst it is encouraging that changes have been initiated, it may understandably leave the complainant somewhat confused. If the complaint is not valid then why amend existing practice?

For example, one complainant [3945] – a mother with an acutely ill baby - outlined a series of (in) actions and miscommunications in the response of the attending Health Visitor. The complaint-response upholds the actions of the Health Visitor, does not apologise for the Health Visitor’s actions but subsequently proffers ‘unreserved apologies for *any* distress this *may* have caused’ on their behalf. Thus, the complaint-response imputes the cause of the distress to the subjective (and presumably factually inaccurate) perceptions of the complainant, rather than the actions of the Health Visitor. Of greater note however, in the absence of the complaint being upheld, is the admission of the potential development of further training in this area and subsequent staff learning:

*As a result of receiving your letter we are reviewing Health Visitor training in relation to infants presenting with symptoms of acute illness,* ***staff will learn from this event’****. [3945]*

A further six complaint-responses did not uphold complaints but then went on to outline changes to practice.

Whilst rebutting complaints yet amending existing practice may appear incongruous and somewhat innocuous, the complaint-responses evidenced an arguably more strident approach to any potential accusations of negligence.

#### Negligence: opposing frames of reference

Acute healthcare is admittedly a complex and challenging undertaking. It is therefore unsurprising that the data corpus of 50 complaints included 12 complex complaints, four deaths and nine apparent accusations of negligence [40]. Complainants perhaps understandably considered ‘care’ and ‘treatment’ to be synonymous with one another [3] whilst complaint-responses clearly delineated each aspect at the outset of the complaint. Indeed ‘treatment’ was often asserted to be ‘medically appropriate’ in responses even when this was not included in the initial complaint. Most notably in one instance, treatment was also considered ‘medically appropriate’ when a hairline fracture was missed on three separate occasions. Whilst this interesting finding may be rooted in the complaint-responders heightened sensitivity to possible negligence claims, it is further evidence of diametrically opposed frames of references e.g. complainant (care) and complaint-responder (clinical treatment). We therefore, conclude our findings with an example of a complaint which references medical negligence and, in our view, embodies the chasm that exists between complainant and complaint-response.

Example 3928 is a daughter complaining on behalf of her mother (the patient) and her father in a relatively succinct two-page letter with a two and half page reply. The patient was treated in a specialist regional oncology centre and then transferred back to the local acute hospital. The daughter noticed the mother was particularly drowsy upon their first visit and checked the medication chart at the bottom of her bed only to find differences in drug dosage and times for one particular medication, which she drew to the attention of the nurse in charge. A nurse duly called the following day to inform the father that *they* had discovered his wife had been on the wrong medication and this was now resolved.

Two further medication problems were also highlighted in the complaint letter. On one occasion the patient was missed from the drugs round and had to ask for her medication whereupon she apparently received a conversational idiom in reply *‘it’s a good job you’re on the ball*!” – this phraseology being noted to occur at times of disaffiliation [41]. A further occasion was identified where the nurse had dispensed the incorrect medication and amended this when challenged by the patient. Thereafter, the daughter submitted a written complaint specifying medical negligence and using a series of extreme case formulations [42] and categorical or high modal items to infer the degree of concern and grievance.

*I find it* ***grossly negligent*** *…* ***too many*** *mistakes … ‘****totally unacceptable’****. I would like you to investigate this and establish how this* ***medical negligence*** *has occurred.’ [3928]*

The use of modals generally marks the speakers’ judgment of the probabilities or the obligations involved in what they are saying. Modality is also related to the speaker’s attitude or opinion regarding the proposition in a clause. The use of *would like* here expresses volition, as the complainant creates an implicit obligation for the reader to take action [43].

The complaint-response is as follows: *“We aim to deliver a high standard of service and I am sorry that on this occasion*

***you felt*** *information provided in* ***your letter*** *and that from staff statements I have found that* ***your mother’s clinical management was appropriate.*** *However, there certainly are times that the service [*sic*] I would have expected your mother to have received* ***has fallen short*** *and for this I offer my sincere and unreserved apologies. A mix up of your mother’s medication was discovered by* ***you and your father*** *and* ***you consider*** *this to be grossly negligent. The doctor* ***inadvertently prescribed X drug 20 mg twice daily instead of once daily.*** *[3928].*

After asserting a threshold of care, the complaint-response starts with an apology (*I am sorry*), not for any wrong-doing but for the (subjective) feelings of the complainant (*you felt*) and quickly bookends this with a rebuttal of any wrong-doing.

The complainant identifies three acts or omissions with medications, one resulting in double the dosage of a medication being prescribed. However, the complaint-response attests that ‘clinical management’ was appropriate with the corollary and minimizer that the service had ‘fallen short’ and a subsequent ‘sincere and unreserved apologies.’ The contradictions in this passage are self-evident. Given the errors identified, agreed (and subsequently unreservedly apologized for) how could clinical management be appropriate? Moreover, the minimizer ‘fallen short’ is somewhat conspicuously disproportionate when placed alongside ‘sincere and unreserved apologies’. Finally, the complaint-response continues in ascribing unintentionality in referring to the doctor ‘inadvertently’ (by accident) making the key prescription error. It is possible that the use of the term ‘inadvertently’ downplays the prescription error or constructs it as an error not requiring apology or redress because of its ‘inadvertant’ nature. The following passages similarly assert the agreed error(s) as accidental or unintentional.

*‘Dr X advised that your mother would not have suffered any adverse effects on 20mg twice daily* ***as the maximum dose that can be given is 60mg****.’ ‘****the inadvertent increase*** *in the dose of X was due to human error and* ***was not responsible for the symptoms of*** *… .’ [3928]*

*‘The investigation identifies two occasions when medication … ’. [not given] ‘****From our records no other medication times were missed and all drugs were signed for****.’ [3928]*

In short, the complaint-response agrees to an accidental/without due care and attention increase in the prescribed dose but clarifies as per de facto negligence – harm did not accrue. Notably, the complaint handler asserts that the patient could theoretically have been mis-prescribed three times as opposed to just two times the amount and (still) not have been harmed as a result. This statement – that there was apparently some leeway with regards to correctly prescribing the drug – may be an attempt to assuage concerns. Nevertheless, it could also be seen to attempt to deflect attention from the mistake that was made and thus avoid an expression of culpability or an apology. Presumably, the statement that these errors did not happen again (beyond the three instances identified) and that all subsequent documentation was appropriate, may also seek to be reassuring. However, the assurance here that no *further* errors were made appears to perhaps neutralise or detract from the errors that *were* made. In tandem with the previous utterances, this statement, therefore, somewhat ironically, potentially adds insult to presumably ‘inadvertent’ injury.

## Discussion

The complaint-responses in our data corpus consistently failed to fully acknowledge the perceived failures in the human relations of healthcare and any subsequent fall-out reported by the complainants. In our previous analysis of the written complaints [3] we observed that complainants reported experiencing visceral emotions, the protracted nature of complaining, including the personal detail and ‘work’ involved in making the complaint, and the cumulative distress. Conversely, the complaint-responses reported here mostly demonstrated a complete lack of alignment with that frame of reference, instead being predicated on an institutional, defensive response. Similarly, the healthcare complaints literature suggests that patients have high expectations of healthcare [44, 45] anticipate care and compassion as well as clinical treatment [46–48] perceive themselves to be custodial guardians of the NHS [49] and when they are harmed [4] or facing cumulative unresolved issues, a threshold of satisfaction is breached thereby instigating a formal complaint [24, 50]. Accordingly, a complaint is initiated with the primary aim of preventing the same problem re-occuring while seeking accountability [26] along with an explanation and an apology [51].

The consumer complaint literature notes that relationships are more effectively reconciled if an apology is provided and responsibility for a perceived trust violation is acknowledged [52] with Lewicki et al. [53] suggesting there are key elements to the structure of an apology and the extent to which all aspects are addressed determines the completeness of the apology. Thus, apologies should include acknowledgement, an expression of regret, accountability, and an explanation along with repentance, repair and forgiveness. Nonetheless, unlike consumer complaint-responses [54] our data demonstrates that apologies (e.g. complaint upheld) largely occur in response to single-issue administrative complaints with broader care and treatment less likely to draw contrition (e.g. partly upheld/not upheld). Instead, we observed the ‘fauxpology’ - a non-apology or false apology (e.g. I am sorry *you feel*) – an interesting modern phenomenon favoured by celebrities and politicians among others [55]. Classically, the fauxpology imputes the cause of distress to the subjective (and possibly misguided) impressions of the complainant, implying that their feelings do not align with the facts, and in this way evades blame or responsibility for the complainable action.

A number of our complaint-responses appeared to have treated some complaints as potentially litigious especially where duty of care or negligence was framed by the complainant. Nevertheless, instead of simply explaining the term ‘medical negligence’ at the outset thereby providing a context for the subsequent reply, complaint-responses adopted an arguably quasi-judicial approach to rebutting the complaint e.g. ‘*the allegations of negligence against Dr X are refuted.’* and in so doing perhaps exacerbated an already fraught complaint-response.

Notably, justice theory underpins most consumer post-complaint behaviour research with procedural justice (the perceived fairness of complaint handling), interactional justice (the interpersonal treatment of complainant) and distributive justice (the fairness of the outcome) [56]. In contrast, our data does not appear to attend to any aspect of justice theory instead treating complaints as subjective and non-factual whilst adopting a default position of upholding staff accounts. If sub-standard care is self-evident, unintentionality, exceptionality or no previous complaints are cited in mitigation. Moreover, whilst failing to acknowledge ‘harm’ and foregoing accountability and explanations, minimal regret may be expressed (e.g. ‘fallen short’) but there remains the incongruity of significant amendments being made to processes or procedures which were apparently not at fault in the first place.

Given our findings, it is unsurprising therefore, that it has been reported that over half of unresolved (local) complaints are subsequently upheld [57]. In tandem with our data, complainants appear to have grievances that are not being fully addressed or resolved [9]. More recently there have been calls to integrate patient level (complaint) data with broader quality improvement methods [58, 59]. We would question the wisdom of complaint data being aggregated and shoehorned into an already struggling QI agenda, with reporting mechanisms that are arguably awash with reporting data [60, 61]. *Individual* patient complaints are exactly that and perhaps need to be addressed as such.

The Public Health Services Ombudsman (PHSO) [26] recently outlined a framework for a consistent approach towards dealing with complaints and support for ‘frontline staff’. This move is to be welcomed but would arguably benefit from the kinds of empirical insights arising from the independent scrutiny of how complaints are handled in real time, with attention to the crucial importance of language in the construction of complaints, complaint responses, and the reception of these responses by complainants. Research on NHS complaints to date [3, 46] have revealed that the *experience of the complainant* must be brought centre stage and afforded more respect and attention in the way in which reforms are framed. Local NHS entities may therefore, benefit more from external agencies providing independent education, training and advice in moving towards complaint resolution, as opposed to complaint handling.

There have, for example, been a number of training projects emerging from Conversation Analytical studies recently that have used naturally occurring healthcare interactions as the basis of identifying best practice in healthcare communications and which might serve as a model for using empirical data analysis to inform best practice in the handling of written complaints too [62–64]. The use of audio recorded, video recorded and written data is authentic, valid and evidence-based and has the potential to engage participants as well as demonstrate effective and potentially ineffective communication strategies. It has the potential to offer a completely different perspective on ‘complaints’ that could re-draw the demarcation lines between complainant and complaint handler and finally move towards accepting the perception of harm as equivalent to harm itself.

### Limitations

We obtained a large hetereogenous data corpus in qualitative terms and have provided a nuanced overview of a poorly understood and under-researched area. We could therefore, only selectively illustrate the qualitative analysis with a few examples. Further, this paper is based upon paired-complaints, specifically complaint-responses, both being subjective accounts and second-hand data.

## Conclusion

NHS complaints are known to be reluctantly proffered by its largely grateful consumer base. Nevertheless, when numerous unresolved issues subsequently breach a given threshold, it is incumbent upon the National Health *Service* to appropriately, transparently and humanely address and resolve the complaint, locally. Our previous analysis of the complaints [3] (with which the complaint responses analysed here are paired) offered evidence of complainants’ emotional investment in and reasoned evidence for the complaint. Building upon these findings and in tandem with our analysis above, we argue that complaint-responses need to attend to these aspects from the outset rather than dismissing their portent and arguably imposing their own institutional agenda. Healthcare complaints’ resolution (as opposed to complaint handling) would benefit from applying a linguistic analysis to the text of complaints and by adopting the consumer literatures’ justice-based approach to post-complaint behaviour. Finally, it is imperative that patient complaints do not get lost in a QI agenda: they are individual, important and remind us of the need to fully engage with the patient’s perspective.

## Data Availability

The datasets used and/or analysed during the current study are available from the corresponding. author on reasonable request.
